# Type B Acute Aortic Dissection as a Perioperative Complication after an Endovascular Abdominal Aortic Repair

**DOI:** 10.3400/avd.cr.20-00107

**Published:** 2021-03-25

**Authors:** Kayoko Natsume, Tsunehiro Shintani, Masanori Hayahi, Kazuhiro Ohkura, Yuto Hasegawa, Takumi Ariya

**Affiliations:** 1 Department of Vascular Surgery, Shizuoka Red Cross Hospital, Shizuoka, Shizuoka, Japan; 2 Department of Cardiac Surgery, Shizuoka Red Cross Hospital, Shizuoka, Shizuoka, Japan

**Keywords:** abdominal aortic aneurysm, type B aortic dissection, endovascular aneurysm repair

## Abstract

Type B aortic dissection (TBAD) is a rare but catastrophic complication of endovascular aneurysm repair (EVAR). We report two cases of TBAD occurring in the perioperative period of EVAR. The intraoperative and postoperative courses were unremarkable. Routine postoperative computed tomography angiography (CTA) revealed TBAD. Conservative treatment was successful, and no adverse aortic events occurred. TBAD that occurs in the perioperative period is likely to be iatrogenic in origin, uncomplicated, and managed with medical therapy: its prognosis is better than when the condition develops in the midterm postoperative period.

## Introduction

Endovascular aneurysm repair (EVAR) is an established treatment for abdominal aortic aneurysm (AAA) in patients with favorable anatomy. However, early complications and the long-term durability of the repair are major limitations of the procedure. Type B aortic dissection (TBAD) is a rare but potentially catastrophic complication of EVAR. Several reports on TBAD after EVAR are available, including cases in which the condition developed in the perioperative or midterm postoperative periods.^1–4,6–9)^ Herein, we described two cases of TBAD during the perioperative period of EVAR and our review of the literature related to this condition and analysis of the outcomes and treatment of perioperative EVAR.

## Case Reports

### Case A

**Figure 1:**
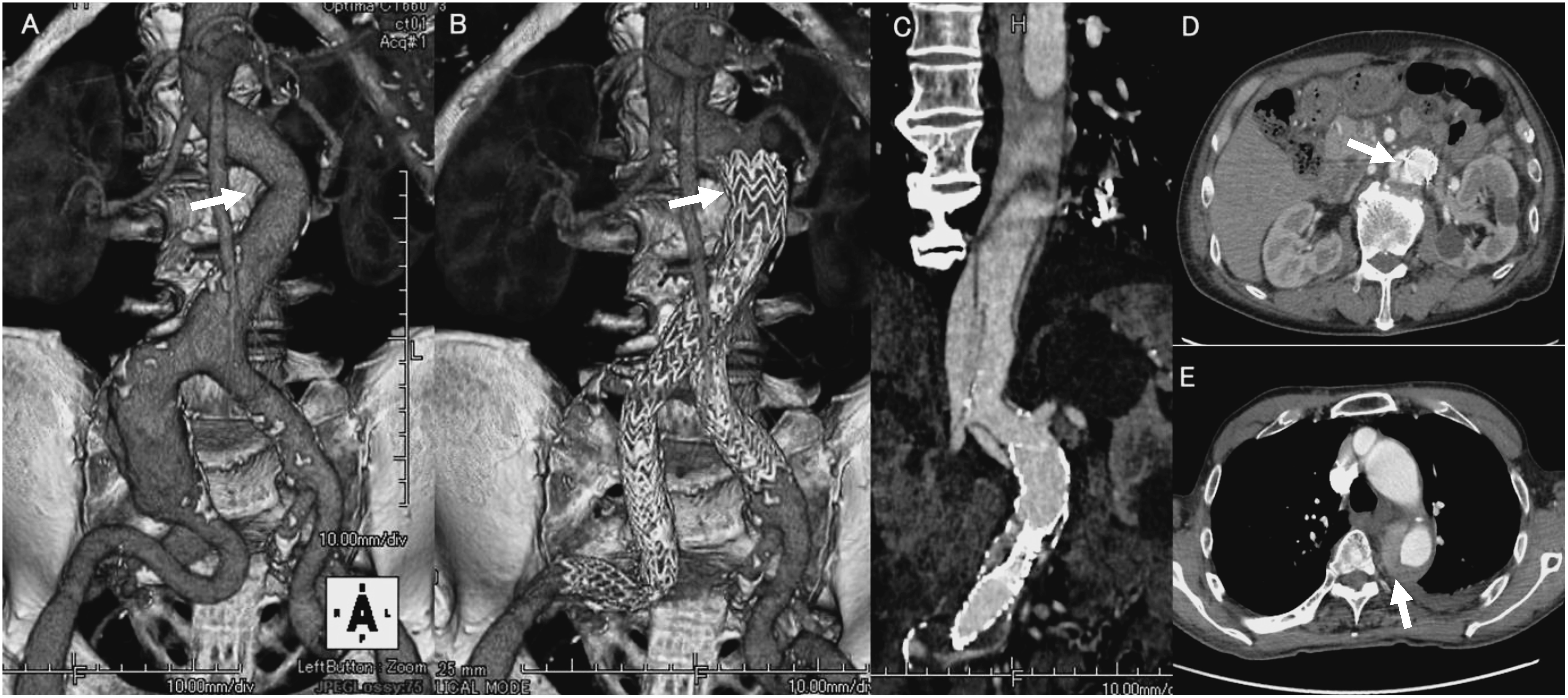
Fig. 1 (**A**) Preoperative three-dimensional computed tomography angiogram (3DCTA) revealing no calcification or atherosclerosis in the proximal neck. It was found that the infrarenal aortic neck angle was 84° (white arrow). (**B**–**E**) Postoperative CTA. (**B**) 3DCTA showing that the proximal edge of the stent graft was located at the angulated neck (white arrow). (**C**) Multiplanar reconstruction revealing type B aortic dissection. (**D**) The axial plane at the proximal edge level of the stent graft. The white arrow shows the dissection entry. (**E**) The axial plane at the level of the left subclavian artery. The white arrow shows that the false lumen was patent but the contrast medium was diluted.

A 78-year-old man was diagnosed with an asymptomatic right common iliac artery aneurysm (40 mm) and dilated abdominal aorta (35 mm) as an incidental finding during follow-up for prostate carcinoma. It was found that the suprarenal and infrarenal aortic neck angles were 60° and 84°, respectively. The proximal aortic neck was 23 mm in diameter proximally, 21 mm in diameter distally, and 40 mm in length with no calcification or atherosclerosis observed (**Fig. 1A**). EVAR was performed under general anesthesia. Coil embolization of the right internal iliac artery and EVAR were performed using a GORE EXCLUDER® (26×14.5×120 mm, 14.5×140 mm, 16×115 mm, 20×95 mm; W. L. Gore and Associates Inc., Flagstaff, AZ, USA) (**Fig. 1B**). After the arterial puncture, a pigtail catheter was advanced through a Radifocus® Guidewire (Terumo Medical, Tokyo, Japan) and exchanged with Amplatz Ultra-Stiff Wire Guides® (Cook Medical, Bloomington, IN, USA) to deploy the stent graft. The tip of the guidewire was positioned at the ascending aorta during EVAR. Stent graft oversizing at the proximal landing zone was 18%. A balloon dilatation at the proximal portion of the stent graft was performed using the Gore® Molding & Occlusion Balloon (W. L. Gore and Associates Inc., Flagstaff, AZ, USA) under nominal balloon pressure. Completion angiography revealed that the stent graft was properly placed, with no endoleak and dissection. We did not observe any hemodynamic changes during EVAR. The systolic blood pressure was controlled at 100–150 mmHg. The postoperative course was uneventful, except for high D-dimer levels (11.2–21.0 µg/ml) at postoperative day (POD) 1–9. Routine postoperative computed tomography angiography (CTA) performed on POD 9 revealed TBAD with a patent false lumen extending from the proximal edge of the stent graft to the origin of the left subclavian artery (**Figs. 1C**–**1E**). The contrast medium was diluted at the origin of the left subclavian artery, suggesting that the dissection was retrograde from the proximal edge of the stent graft. The maximum transverse diameter of the descending thoracic aorta enlarged from 32 to 42 mm, with a false lumen diameter of 17 mm. No migration or collapse of the stent graft was detected. Next, he was medically treated. The systolic blood pressure was controlled at <120 mmHg using intravenous and transdermal antihypertensive drugs. He was discharged with no sequelae. The 1-year follow-up CTA examination revealed no expansion of the aorta and false lumen.

### Case B

**Figure 2:**
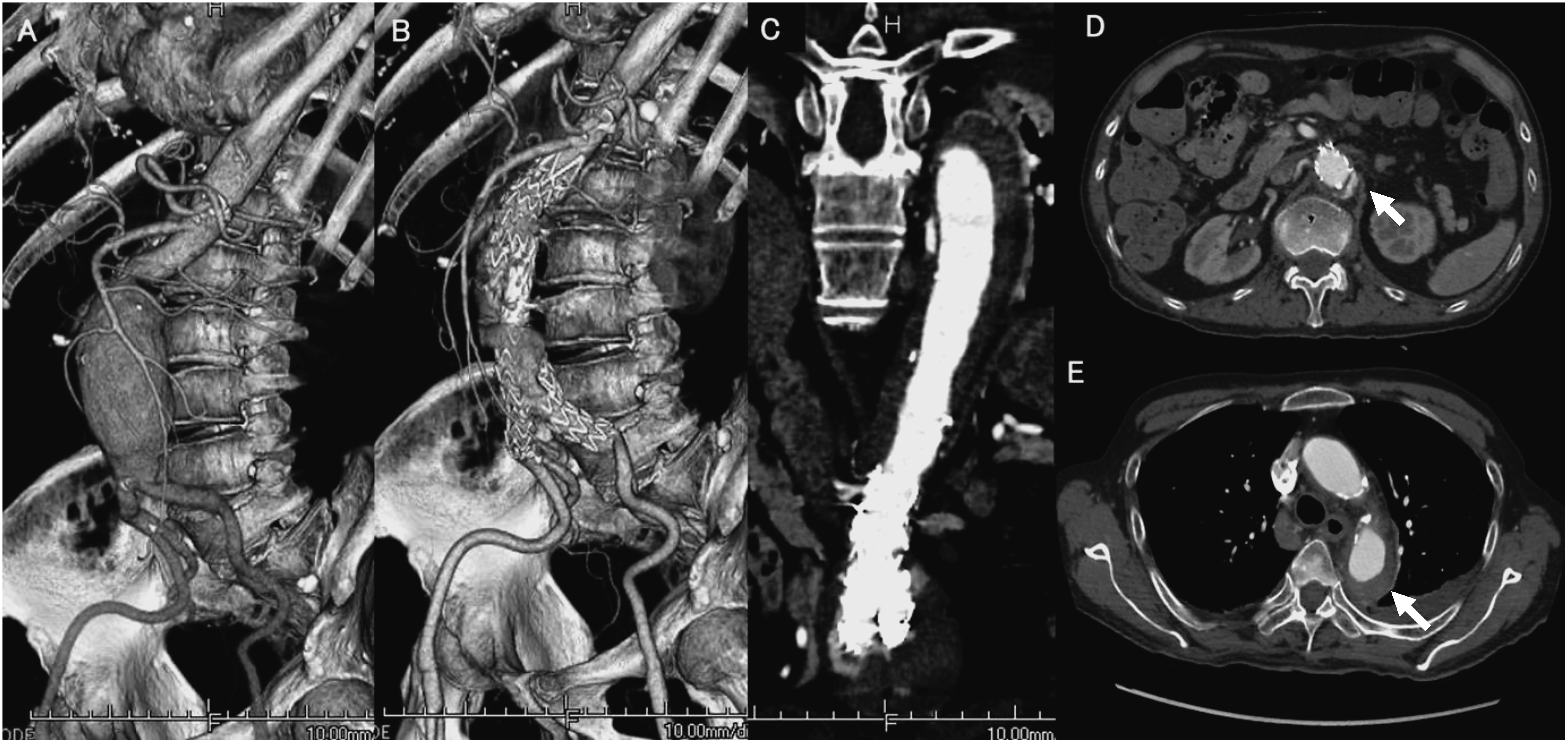
Fig. 2 (**A**) Preoperative three-dimensional computed tomography angiogram (3DCTA) revealing no calcification or atherosclerosis in the proximal neck. (**B**–**E**) Postoperative CTA. (**B**) 3DCTA showing the stent graft with proximal bare stents. (**C**) Multiplanar reconstruction revealing type B aortic dissection. (**D**) The axial plane at the proximal edge level of the stent graft. The white arrow shows the dissection entry. (**E**) The axial plane at the level of the left subclavian artery. The white arrow shows the thrombosed false lumen.

An 81-year-old man was diagnosed with an asymptomatic AAA (63 mm) as an incidental finding during examination of malignant lymphoma. He previously received percutaneous coronary intervention for angina pectoris and chemotherapy for malignant lymphoma. CTA revealed that the suprarenal and infrarenal aortic neck angles were 30° and 60°, respectively. The infrarenal aortic neck was 19 mm in diameter proximally, 17 mm in diameter distally, 15 mm in length with no calcification, and atherosclerosis observed (**Fig. 2A**). EVAR was immediately performed under general anesthesia after chemotherapy using an Endurant II (23×16×145 mm, 16×16×82 mm, 16×16×124 mm; Medtronic Cardiovascular, Anta Rosa, CA, USA) (**Fig. 2B**). After the arterial puncture, a pigtail catheter was advanced through the Radifocus® Guidewire (Terumo Medical, Tokyo, Japan) and exchanged with Amplatz Ultra-Stiff Wire Guides® (Cook Medical, Bloomington, IN, USA) to deploy the stent graft. The tip of the guidewire was then positioned at the ascending aorta during EVAR. Stent graft oversizing at the proximal landing zone was 20%–28%. The reverse slider technique was used for adequate proximal sealing.^5)^ A balloon dilatation at the proximal portion of the stent graft was then performed using Gore® Molding & Occlusion Balloon (W. L. Gore and Associates Inc., Flagstaff, AZ, USA) under nominal balloon pressure. Completion angiography revealed that the stent graft was properly placed, with no endoleak and dissection. We did not observe any hemodynamic changes during EVAR. The blood pressure was perioperatively controlled at 90–150 mmHg. The postoperative course was normal, except for extremely high levels of fibrinogen degenerative products (FDPs, 138–176 µg/ml) detected on POD 1–6. Routine postoperative CTA on POD 6 revealed TBAD with a partially thrombosed false lumen extending from the proximal edge of the stent graft to the origin of the left subclavian artery (**Figs. 2C**–**2E**). The proximal end of the false lumen was thrombosed, suggesting that the dissection was retrograde from the proximal edge of the stent graft. The maximum transverse diameter of the descending thoracic aorta enlarged from 28 to 37 mm, and the false lumen diameter was 12 mm. No migration or collapse of the stent graft was detected, and he was medically treated. Systolic blood pressure was controlled at <120 mmHg using intravenous and transdermal antihypertensive drugs. He was discharged with no sequelae. CTA performed at the 1-year follow-up revealed no expansion of the aorta, and the false lumen diameter reduced to 10 mm.

## Discussion

The risk of aortic dissection after thoracic endovascular aortic repair (TEVAR) has been reported to be 2.4%.^7)^ However, TBAD after EVAR is a rare complication, with a reported risk of 0.5% estimated at a single-center study.^8)^ Although the exact mechanism underlying the development of EVAR has not yet been elucidated, spontaneous type B dissection and iatrogenic dissection are proposed to be two major etiologies (**Table 1**). Iatrogenic dissection should be considered when dissection occurs during the perioperative period. Factors that contribute to the development of iatrogenic dissection after EVAR are classified to be either procedure- or device-related. Balloon dilatation, wire manipulation, and oversizing the stent graft are procedure-related factors implicated in the condition. The use of a device with proximal bare stents is a device-related factor that influences TBAD occurrence. A hostile aortic neck including aortic calcification or an irregularly shaped or highly angulated infrarenal aortic neck is closely associated with both the procedure- and device-related factors.^1,4,7)^ We found five reported cases of TBAD occurring within 2 weeks after EVAR (cases 1, 6, 8, 10, and 20 in **Table 1**). All cases were suspected to be classified as iatrogenic in etiology. However, in case 20, the cause of dissection was suspected to be the guidewire manipulation during percutaneous coronary intervention immediately following EVAR. In cases 6 and 8, the exact cause of dissection and the entry site were not clearly observed. In cases 1 and 10, the etiology was clearly observed, and the dissection was suspected to be retrograde from the proximal edge of the stent graft.

**Table 1:** **Table 1** Summary of case reports of type B aortic dissection after an endovascular abdominal aortic aneurysm repair

Case	First author (year)	Device	Time after EVAR	Etiology	Complication	Entry	Treatment	Outcome
1	Girardi (1999)^1)^	Handmade Palmaz	2 days	Iatrogenic Calcified neck	None	Level of renal artery	Medication→open repair	Alive
2	Haulon (2003)^2)^	Excluder (Gore)	5 months	Spontaneous	Rupture	Unknown	Medication	Dead
3	Keulen (2009)^2)^	Talent (Medtronic)	2 years	Spontaneous	Collapse	Distal of LSCA	TEVAR	Alive
4	Iyer (2009)^2)^	Zenith (CooK)	11 weeks	Spontaneous	Compression	Distal of LSCA	TEVAR/thrombectomy	Alive
5	Puli (2011)^3)^	Zenith (CooK)	2 years	Spontaneous	Compression	Distal of LSCA	TEVAR	Alive
6	Tolenaar (2011)^4)^	Endurant (Medtornic)	2 days	Iatrogenic Unclear	None	Unknown	Medication→flap fenestration	Alive
7	Khanbhai (2013)^3)^	Zenith (CooK)	6 weeks	Spontaneous	None	Unknown	Medication	Alive
8		Endurant (Medtornic)	1 day	Iatrogenic Unclear	None	Unknown	Medication	Alive
9		Talent (Medtronic)	3 weeks	Spontaneous	None	Level of celiac artery	Medication	Alive
10	Mamopoulos (2013)^3)^	Endurant (Medtornic)	2 days	Iatrogenic Proximal bare stent	None	Level of suprarenal stent	Medication	Alive
11	Yamamoto (2013)^3)^	Zenith (CooK)	1 year	Spontaneous	Rupture	Level of suprarenal stent	Medication	Dead
12	Psacharopilo (2014)^3)^	Excluder (Gore)	1 year	Spontaneous	Collapse	Unknown	Axillofemoral bypass	Dead
13	Vainas (2014)^2)^	Zenith (CooK)	10 years	Spontaneous	Collapse	Des. Ao	Open repair/TEVAR	Alive
14	Sirignano (2015)^3)^	Endurant (Medtornic)	4 weeks	Spontaneous	TAA expansion	Unknown	TEVAR	Alive
15		Excluder (Gore)	2 years	Spontaneous	None	Unknown	Medication	Alive
16	Yoshiga (2015)^2)^	Endurant (Medtornic)	6 months	Spontaneous	Collapse	Distal of LSCA	TEVAR	Alive
17	Daniel (2016)^2)^	Endurant (Medtornic)	1 year	Spontaneous	Rupture	Unknown	Open repair	Dead
18	Goto (2017)^2)^	Excluder (Gore)	2 years	Spontaneous	Collapse	Unknown	Axillofemoral bypass	Alive
19	Jayakumar (2017)^6)^	Zenith (CooK)	5 months	Spontaneous	Compression	Distal of LSCA	TEVAR	Alive
20	Park (2017)^7)^	Endurant (Medtornic)	1 week	Iatrogenic PCI	Malperfusion	Distal of LSCA	Medical→TEVAR	Alive
21	Shintani (2017)^8)^	Endurant (Medtornic)	2 years	Spontaneous	Expansion	Unknown	EVAR	Alive
22	Itoga (2018)^2)^	Excluder (Gore)	4 years	Spontaneous	Collapse	Des. Ao	TEVAR	Alive
23	Nomura (2019)^2)^	Zenith (CooK)	7 years	Spontaneous	Rupture	Des. Ao	Medication	Dead
24		Excluder (Gore)	6 months	Spontaneous	Collapse	Distal of LSCA	TEVAR	Dead
25	Ostapenko (2019)^9)^	Zenith (CooK)	Several years	Spontaneous	Collapse	Distal of LSCA	TEVAR	Dead
26	Rodriguez (2019)^3)^	Zenith (CooK)	4 weeks	Spontaneous	Compression	Distal of LSCA	TEVAR	Alive

EVAR: endovascular abdominal aortic aneurysm repair; PCI: percutaneous coronary intervention; LSCA: left subclavian artery; Des. Ao: descending aorta; TBAD: type B aortic dissection; TEVAR: thoracic endovascular aortic repair

In the present report, stent grafts were implanted without complications, and oversizing of the stent grafts was within the manufacturers’ recommendations in case A, and the stent graft at the proximal landing zone was partially oversized in case B. Our case, i.e., case A, demonstrated a highly angulated infrarenal aortic neck, and the proximal edge of the stent graft was located at the angulated neck. Our case B was implanted with a device with proximal bare stents. Moreover, the reverse slider technique might provide great stress on the proximal neck. CTA findings suggested that the dissection was retrograde from the proximal edge of the stent graft. Therefore, our cases are considered to be iatrogenic in etiology.

Our cases were asymptomatic, except abnormalities related to blood coagulation. Although EVAR is generally associated with the activation of the coagulation and fibrinolytic pathways, our cases exhibited extremely high levels of biomarkers compared with previously reported cases of perioperative EVAR (with reported D-dimer levels of 0.32–0.43 µg/ml and FDP levels of 8.6–12.0 µg/ml).^9)^ Blood coagulation abnormalities may indicate TBAD after EVAR.

The treatment for TBAD after EVAR is decided depending on the presentation. In the literature, TBAD, except case 20 that developed during the perioperative period (within 2 weeks of EVAR) were uncomplicated and initially managed with medical therapy (**Table 1**). In case 6, endovascular fenestration of the intimal flap was performed due to severe pain after medical therapy and fear of stent graft collapse. Although this fenestration was unsuccessful, the patient was finally managed with medical therapy. All other cases had successful medical therapy and were alive. Case 20 had antegrade dissection from the distal arch of the left subclavian artery to the proximal stent graft and was complicated with malperfusion treated with TEVAR. Conversely, most cases (17/20) of TBAD during the midterm postoperative period (2 weeks after EVAR) were complicated, including malperfusion, aneurysm expansion, or aneurysm rupture, which might require surgical or endovascular interventions.^2,3,6,8,10)^ Majority of these complicated cases had antegrade dissection from the distal arch of the left subclavian artery to the proximal stent graft.

Finally, the prognosis is different depending on the onset of TBAD after EVAR. All cases of TBAD that developed during the perioperative period survived. In case 1, acute setting was successfully managed with medical therapy; however, the expansion of thoracic aorta during the chronic stage (at 4 months after EVAR) required open repair. In contrast, seven deaths occurred due to TBAD during the midterm postoperative period. Nomura et al. reviewed 10 cases of spontaneous complicated TBAD after EVAR and found that collapse of stent graft and thrombosis occurred in 80% of these cases and suggested that they are fatal because the reentry is not formed by the presence of the stent graft.^2)^

## Conclusion

In summary, we have experienced two cases of TBAD occurring in the perioperative period of EVAR. The intraoperative and postoperative courses were unremarkable, except abnormalities related to blood coagulation. Blood coagulation abnormalities may indicate TBAD after EVAR. Conservative treatment was successful, and no adverse aortic events occurred. According to the literature, perioperative TBAD originating from the device is generally retrograde and does not malignantly behave, whereas those caused by guidewires or spontaneously occurring during the follow-up period are antegrade and have poor outcomes because reentry is not formed by the presence of the EVAR device. However, to reduce perioperative TBAD, EVAR procedure should be more carefully performed in patients with hostile aortic neck or when device with proximal bare stents is used. TBAD after EVAR is rare, and further accumulation and publication of cases is necessary to elucidate the characteristics of the condition.
